# Virulent Avian Infectious Bronchitis Virus, People’s Republic of China

**DOI:** 10.3201/eid1812.120552

**Published:** 2012-12

**Authors:** Jinling Feng, Yanxin Hu, Zhijun Ma, Qi Yu, Jixun Zhao, Xiaodong Liu, Guozhong Zhang

**Affiliations:** Author affiliations: The Key Laboratory of Animal Epidemiology and Zoonosis, Ministry of Agriculture, China Agricultural University, Beijing, People’s Republic of China (J.L. Feng, Y.X. Hu, J.X. Zhao, G.Z. Zhang); Animal Husbandry and Veterinary Station of Beijing, Beijing (Z.J. Ma, Q. Yu, X.D. Liu)

**Keywords:** Avian infectious bronchitis virus, virulence, pathogenicity, tissue tropism, phylogenetic analysis, viruses, People’s Republic of China, chickens

## Abstract

A virulent avian infectious bronchitis virus (IBV) was isolated from 30-day-old broiler chickens that exhibited respiratory symptoms, nephropathologic lesions, and a high proportion of deaths in the People’s Republic of China during 2005. The strain, designated YN, was genetically and pathologically characterized. Phylogenetic analysis showed that YN and most of the previously characterized IBV isolates found in China were phylogenetically classified into 2 main genetic clusters. The YN isolate caused severe lesions and resulted in deaths of 65% in experimental infections of 30-day-old specific-pathogen–free chickens. Tracheal and severe kidney lesions developed in all infected birds, confirming the ability of YN strain to induce both respiratory and renal disease. IBV antigens were detected by immunohistochemical analysis in the trachea, lung, kidney, and bursa, consistent with histopathologic observations, virus isolation, and reverse transcription PCR detection. We showed that YN IBV exhibits severe pathogenicity in chickens, and that similar viruses are prevalent in China.

## Abstract 2

YN strain shows severe pathogenicity in chickens.

Avian infectious bronchitis virus (IBV), a member of family *Coronaviridae,* order Nidovirales, causes a highly contagious respiratory and sometimes urogenital disease of chickens that is characterized by respiratory signs, nephritis, or reduced egg production and quality in layer chickens. IBV is a major poultry pathogen that is endemic worldwide and leads to serious economic losses.

The main method of protecting poultry from infectious bronchitis (IB) is the administration of live or killed vaccines. However, IB continues to cause economic losses in the poultry industry despite intensive vaccination programs in many countries (*1*–*5*). Outbreaks of IB often are due to infections with strains serologically different from those used for vaccination (*2*,*6*). Since IBV was first described in 1931, a large number of serotypes or variants have emerged, and some have become endemic worldwide (*4*,*7*–*12*). That little or no cross-protection occurs between different serotypes of IBV is well known (*13*). Therefore, continuous determination of the epidemic serotype and production of new generations of vaccines are crucial for controlling IB in each geographic region or country.

IBV was first isolated in the People’s Republic of China in the early 1980s; since then, the Massachusetts-type (e.g., H120, H52, Ma5, W93, and 28/86) or Connecticut-type live attenuated IB vaccines and the inactivated killed oil-emulsion vaccine have been used to prevent and control the disease. However, the serotypes of the vaccines used have been epidemiologically determined to differ from those of the predominant IBV isolates in China that form 2 large groups of unique strains, named A2-like and QXIBV-like strains (*5*,*6*,*14*–*18*). Therefore, because of the lack of a vaccine against endemic strains of IBV in China, IBV infection has remained a problem in the Chinese poultry industry.

We isolated a virulent IBV strain from 30-day-old broiler chickens in the Yunnan Province and characterized it using sequence alignment, phylogenetic analysis, pathogenicity studies, histopathologic observation, and immunohistochemical (IHC) examination. The results indicate that the isolate is genetically similar to most of the prevalent strains of IBV found in China. We also showed that IBV YN isolate displays more severe pathogenicity than previously characterized strains in China.

## Methods

### Virus and Animals

The YN strain was isolated in 2005 from 30-day-old broilers that had been inoculated oculonasally with H120 vaccines of IBV on days 1, 7, and 21, respectively. The sick birds had respiratory signs, severe renal disease, and a death rate of 30%. The virus was propagated in 10-day-old specific-pathogen–free (SPF) embryonated chicken eggs at 37°C for 40 h. Allantoic fluid was recovered from infected eggs and stored at −80°C. All animal research was approved by the Beijing Administration Committee of Laboratory Animals under the leadership of the Beijing Association for Science and Technology (approval ID is SYXK [Beijing] 2007–0023).

### Viral RNA Extraction, Reverse Transcription PCR, and DNA Sequencing

On the basis of IBV nucleotide sequences (M41, A2, BJ, ZJ971, and SC021202; GenBank accession nos. DQ834384, AY043312, AY319651, AF352313, and AY237817, respectively), 22 pairs of specific primers (Table 1) were designed to amplify the complete genome (excluding the 5′- and 3′-terminal segments) of the YN strain. Viral RNA was extracted from virus-infected allantoic fluid by using Trizol Reagent (Invitrogen, Carlsbad, CA, USA) according to the manufacturer’s instructions. Reverse transcription (RT) was performed at 37°C for 1 h by using 3 μg total RNA, 1 μL random primers (500 μg/mL, random hexadeoxynucleotides; Promega, Madison, WI, USA) and 0.5 μL M-MLV RT (200 U/μL) (Promega). For PCR, 1 U Taq DNA Polymerase (Promega) and 10 pmol of each primer were added to 100 ng cDNA as template in a total of 20 μL reaction volume. PCR was performed at 95°C for 5 min, followed by 35 cycles of denaturation (95°C, 45 s), annealing (53°C or 55°C, 45 s), and polymerization (72°C, 2 min), and the postpolymerization step was performed at 72°C for 10 min. Amplified sequences were analyzed by 1.2% agarose gel electrophoresis.

**Table 1:** Primers used to amplify the complete genomic sequence of avian infectious bronchitis virus YN strain, People’s Republic of China

Primer*	Location, bp	Upstream primer	Downstream primer	Length, bp
1	22–1,475	ATATCATACATACTAGCCTTG	AGTTAAGTCGTTTCGCATG	1,454
2	1,302–2,411	CCAACTGGTTTTATGGGTGC	TGGAAGTGTCACTGCCTCG	1,110
3	2,240–3,784	ACTTGGTAGAGTTTCTGGGG	TTGACATACGAAGGTGTGACA	1,545
4	3,635–5,161	TGTAAACGCCGCAAATGAG	GGCAACTTGGAATCCTCTT	1,527
5	5,040–6,636	GATCTTACTGACTTTGAAC	CACTGAAACACTTAACTG	1,597
6	6,424–7,936	GGAATTGTGACGAGTATGG	TGAAAGTATACCCTATGAGGA	1,513
7	7,746–9,246	GAAATTGTCGGTTACACTCAG	TAGAACGCATAGTAACAGGG	1,501
8	9,130–9,985	CTAACGCTGAAACTCCA	GTTGGCAATAGGAAAGTA	856
9	9,082–10,645	TCAGTAGGCGGATAAAAGG	ATAGGCAACACACGGTCG	1,564
10	10,516–12,092	ATTTCAAAGTTTCGGTTGACC	CACAGAAGCTCCTCCATAG	1,577
11	11,861–13,471	GTTTTACAATCTAAAGGTCAT	AAGAGCGGGATCTCCTAC	1,611
12	13,270–14,908	CAACATTCTTTTCTCTACAC	GATATAACGCTCCATAACT	1,639
13	14,712–16,138	CTGATTCTAAGTGTTGGGTTG	TCCTTTGAGGTACTATGCGA	1,427
14	16,027–17,539	TGCTCGTGTTGTTTTTACTGC	CACTTGCTCCTTGCCTATTTT	1,513
15	17,362–18,993	CCACTTGAGGGCTTTGT	TAAACATACAGGTTCGCT	1,632
16	18,945–20,442	AAGCGGTATYCNTATGTAGAA	ATAGTRCAVACAAAAKRGTCA	1,498
17	20,336–21,920	ACTGAACAAAAGACMGACTT	CCACCAGAAACTACAACT	1,585
18	21,873–22,919	AAGGTTAATCCCTGTGAAG	AGTYTCVGTAAGAATAGCA	1,047
19	22,723–23,837	CTTTTGCHACTCAGATDCA	AGATTTCTTACCACACTTACT	1,115
20	23,435–24,898	ATTTGTAGAAGATGACGAT	CATTGTTGACCATTAGTTA	1,464
21	24,747–26,141	GCAGCGATAATACTTACCGTG	GCTTGGCGTCTCCAGTATC	1,395
22	25,965–27,650	TATCAAACTAGGAGGACCA	GCTCTAACTCTAAACTAGCCT	1,686

*Primer locations are listed according to strain SC021202 (GenBank accession no. EU714029).

The amplified DNA products were purified by using an AxyPrep DNA Gel Extraction Kit (AxyGEN, Union City, CA, USA), and the purified products were ligated to the pMD18-T Easy Vector system (Promega). Recombinant plasmids were extracted from positive clones by using the E.Z.N.A.R. Plasmid Miniprep Kit (Omega, Norcross, GA, USA) and identified by *Eco*RI restriction digestion (Promega). Nucleotide sequencing reactions were performed by Sunbio Biotech (Bejjing, China).

### Sequence and Phylogenetic Analysis

Complete genome or S1 gene sequences of IBV were obtained from GenBank, and these IBV sequences and the complete coding sequence of the IBV YN isolate were aligned and analyzed by using the ClustalW multiple alignment algorithm in the MegAlign program of the DNASTAR software suite (version 3.1; DNAstar, Madison, WI, USA).

The phylogenetic tree of S1 gene or complete genomic sequences was constructed by using MEGA4.0 software (www.megasoftware.net) by the neighbor-joining method (1,000 bootstrap replicates). Evolutionary distances were computed by the pairwise distance method using the maximum composite likelihood model (*19*).

### Clinicopathologic Assessment in Chickens

Forty 30-day-old SPF white leghorn chickens were randomly divided into 2 groups of 20 birds each. All birds in 1 group were challenged intranasally with at least 10^5^ 50% egg infectious dose per 0.2 mL of IBV YN strain. Birds in another group were inoculated with 0.2 mL phosphate-buffered saline (PBS) as noninfected controls. Birds were housed in isolators and provided feed and water ad libitum.

All birds were observed daily for signs of disease (e.g., disheveled feathers, depression, respiratory signs, or diarrhea) and death for 21 days. Gross pathologic changes were observed, and tissues (trachea, lung, kidney, bursa, liver, and brain) were collected for RT-PCR from 10 randomly selected chickens that died within 4–10 days after inoculation, virus isolation, and histopathologic and IHC analyses. Serum samples collected from birds that survived were tested for IBV antibodies by a commercial ELISA kit (IDEXX Laboratories, Westbrook, ME, USA). The endpoint titers were calculated according to the manufacturer’s instructions, and titers >396 were considered positive for IBV antibody.

### RT-PCR of Tissue Samples

Tissue samples from deceased birds were collected and used for RT-PCR to determine tissue distribution of the virus. Total RNA was obtained by using Trizol Reagent (Invitrogen) as recommended for tissue samples. RT-PCR was performed as described above by using a pair of primers (forward: 5′-TTTTGGTGATGACAAGATGAA-3′; reverse: 5′- CGCATTGTTCCTCTCCTC-3′), which amplify and detect a 403-bp fragment of the S1 gene of IBV. PCR products were analyzed on 1.5% agarose gels.

### Virus Re-isolation from Infected Tissue Samples

Tissue samples collected after challenge were used for virus isolation. Briefly, at least 6 SPF embryos were inoculated through the allantoic cavity, with each sample, containing 10,000 U/mL penicillin and 10,000 µg/mL streptomycin (0.2 mL/egg). The eggs were candled daily, and allantoic fluids from 3 inoculated embryos were collected 48 h after inoculation for RT-PCR amplification. The remaining embryos were examined 6 days after inoculation for characteristic IBV lesions, such as dwarfing, stunting, or curling of embryos.

### Histopathology

Tissues (trachea, lung, kidney, bursa, liver, and brain) were collected and fixed by immersion in 10% neutral formalin at room temperature for 48 h. Tissue was then routinely processed, embedded in paraffin wax, and cut into 5-μm sections. The sections were stained with hematoxylin and eosin and examined by light microscopy for lesions resulting from IBV infection.

### Immunohistochemistry

All sampled tissues were examined by IHC analysis to detect viral antigen. Briefly, 5-μm tissue sections were subjected to antigen retrieval (*20*) and were then incubated in 10% normal goat serum in PBS for 30 min to block nonspecific binding sites. Slides were further incubated with chicken anti-IBV hyperimmune serum at 1:500 dilution in PBS for 2 h, followed by incubation with a horseradish peroxidase–conjugated rabbit chicken IgG for 1 h. The reaction was visualized by the addition of 3,3-diaminobenzidine (DAB; Sigma, St. Louis, MO, USA) for 15 min. After IHC staining, sections were counterstained with hematoxylin, air dried, and examined by light microscopy.

## Results

### Genome Sequencing

The complete genome sequence (excluding the 5′- and 3′-terminal segments) of YN strain was obtained by assembling 22 overlapping sequences that ranged from 856 bp to 1,686 bp by the DNAstar software. The nucleotide sequences were submitted to GenBank under accession no. JF893452. The complete genomic sequences (excluding the 5′- and 3′-terminal segments) of YN isolate comprised 27,635 nt, which putatively contains 6 different genes, each containing single or multiple open reading frames.

### Phylogenetic Analysis

**Figure 1:**
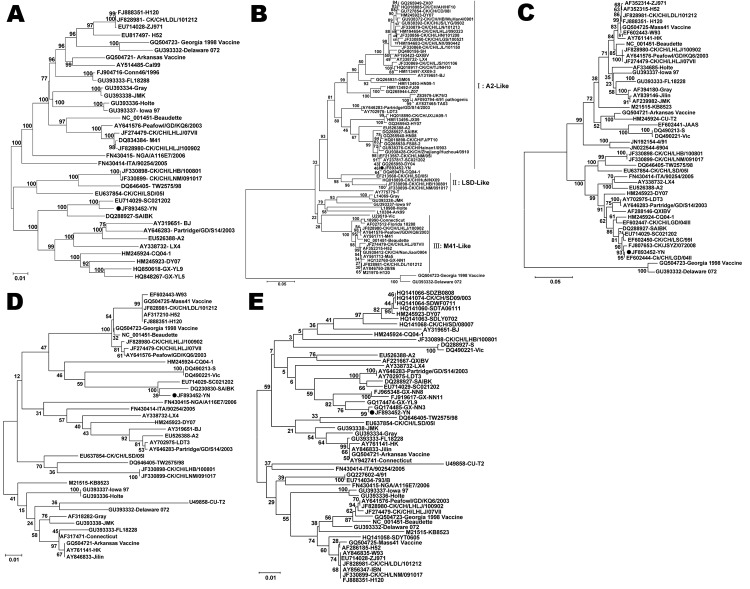
Phylogenetic tree of avian infectious bronchitis virus based on the nucleotide sequences of the complete genome (A), S1 gene (B), S2 gene (C), E gene (D), M gene (E), and N gene (F). The phylogenetic tree was constructed by the neighbor-joining method with 1,000 bootstrap replicates (bootstrap values are shown on the tree). The isolate sequenced in this study is indicated with a black dot.

Phylogenetic trees for the genome and each gene of IBV reinforced the viral nucleotide sequencing results, suggesting that the YN isolate shares an immediate ancestor with the SC021202 strain (Figure 1). To elucidate the phylogenetic relationships of China IBV strains, we further analyzed S1 genes of 70 IBVs, including 51 China isolates and 19 standard strains or vaccine strains (Figure 1, panel B). The data indicated that China isolates could be differentiated into 3 distinct genetic groups or genotypes. Group I included 41 of the 51 field isolates, which were tentatively named A2-like viruses. Four China field isolates were included in group II, which were tentatively named LSD-like viruses. China group III comprised 6 isolates, which were grouped with the Massachusetts serotype. These results showed that at least 3 distinct groups existed in chicken flocks in China, and A2-like and M41-like viruses were mainly responsible for the IB panzootic, consistent with findngs of our previous studies (*5*,*16*).

### Sequence Comparisons

Ten virus sequences were selected for pairwise comparisons with the YN strain according to the phylogenetic analysis for S1 (Figure 1, panel B), which included 2 common vaccine strains in China (M41 and H120) and 8 China isolates from 3 different gene clusters (DY07, LX4, A2, and SC021202 from cluster I; CK/CH/LSD/05I and CK/CH/LHB/100801 from cluster II; CK/CH/LHLJ/07VII and CK/CH/LDL/101212 from cluster III) (Table 2). Overall, the complete genomic sequences (excluding the 5′- and 3′-terminal segments) were 87.1%–98.0% identical among the isolates, and the YN strain had the highest nucleotide identity (98.0%) to the SC021202 strain isolated in southern China (GenBank accession no. EU714029). For each gene, the identity hanged from 93.7% to 100% between YN and SC021202 strains (Table 2).

**Table 2:** Percent sequence identities between the YN isolate and 10 selected IBV strains, People’s Republic of China

Strain*	Gene 1													Gene 2	
	Nsp1	Nsp2	Nsp3	Nsp4	Nsp5	Nsp6	Nsp7	Nsp9	Nsp10	Nsp11	Nsp12	Nsp13		S1†	S2†
DY07	90.3	85.9	88.2	94.8	88.1	92.5	91.3	92.1	89.9	89.2	88.4	91.2		79.2 (77.9)	93.4 (94.3)
LX4	82.2	91.9	97.3	97.6	94.8	100	97.3	97	96.0	96.3	92.3	96.4		82.1 (81.2)	89.7 (92.6)
A2	81.8	89.9	90.2	97.6	92.4	99.1	94.5	98	96.0	89.5	90.0	94.7		91.5 (90.0)	92.7 (94.2)
SC021202	**97.7**	**99.0**	**99.0**	**100**	**100**	**100**	**99.0**	**97.9**	**99.0**	**100**	**100**	**100**		**99.3 (98.6)**	**99.5 (99.5)**
CK/CH/LSD/05I	84.9	92.9	92.5	96.4	94.3	99.1	95.9	96.2	96.5	96.2	94.1	95.4		82.9 (82.6)	89.3 (92.5)
CK/CH/LHB/100801	86.1	94.2	92.2	100	94.3	99.1	95.9	96.6	96.0	96.2	94.4	96.4		82.9 (82.6)	89.3 (92.5)
CK/CH/LHLJ/07VII	86.7	86.1	88.7	93.6	87.3	91.3	90.8	88.4	90.7	89.7	88.3	88.0		69.1 (75.4)	84.7 (87.4)
CK/CH/LDL/101212	84.9	88.2	89.0	95.2	89.5	91.9	91.0	87.6	90.9	89.8	88.0	88.3		80.7 (77.8)	84.6 (87.7)
M41	87.1	93.8	91.5	96.4	95.3	98.2	99.0	96.6	96.3	97.1	92.3	92.1		81.8 (78.2)	86.1 (89.1)
H120	86.6	93.5	90.8	96.4	95.3	98.2	99.0	97.0	96.2	96.7	92.6	91.1		81.6 (78.2)	85.8 (88.8)

	Gene 3				Gene 4		Gene 5			Gene 6		3′ UTR	Genome
	3a†	3b†	3c (E)†		M†		5a†	5b†		N†			
DY07	92.5 (91.2)	76.6 (65.1)	87.8 (88.9)		92.8 (96.9)		92.4 (92.3)	91.2 (85.4)		86.4 (92.2)		96.7	89.5
LX4	93.1 (91.5)	74.1 (66.7)	87.5 (88.2)		91.3 (93.0)		89.9 (87.9)	90 (89.3)		85.9 (90.3)		76.9	87.5
A2	87.4 (80.7)	78.4 (67.2)	87.5 (88.2)		91.9 (93.8)		83.9 (78.8)	91.2 (86.9)		85.9 (90.0)		95.2	87.9
SC021202	**96.6 (93.2)**	**93.7 (93.6)**	**98.2 (98.2)**		**95.6** (**98.7**)		**99.5 (98.5)**	**99.6 (98.8)**		**99.7 (100)**		**98.7**	**98.0**
CK/CH/LSD/05I	84.4 (68.4)	83.7 (70.3)	87.2 (88.2)		89.8 (92.1)		86.5 (81.8)	94.0 (91.7)		88.3 (93,7)		89.4	88.2
CK/CH/LHB/100801	85.2 (84.7)	83.7 (71.9)	87.2 (88.2)		89.7 (93.4)		86.5 (81.8)	94.0 (91.7)		88.1 (91.0)		93.2	87.7
CK/CH/LHLJ/07VII	82.18 (63.2)	82.1 (79.7)	89.0 (88.1)		89.1 (92.9)		81.8 (75.4)	93.6 (89.0)		87.0 (91.2)		98.7	92.5
CK/CH/LDL/101212	81.0 (73.7)	82.1 (78.1)	88.1 (83.5)		89.7 (93.3)		84.9 (80.0)	93.6 (89.0)		87.0 (91.9)		94.6	87.1
M41	82.3 (72.9)	82.6 (79.7)	89.3 (89.1)		89.4 (93.8)		81.4 (74.2)	94.0 (89.3)		88.0 (91.5)		94.2	87.1
H120	81.2 (73.7)	82.6 (78.1)	88.1 (84.5)		89.7 (93.4)		85.5 (80.3)	93.6 (89.3)		87.2 (92.2)		78.9	87.2

*Except for the M41 and H120 strains which are in the IBV vaccine, all other strains are China isolates from different groups. Highest identities are indicated in **boldface**. IBV, infectious bronchitis virus; UTR, untranslated region. †Percentage of amino acid identity is shown in the parenthesis.

**Figure 2:**
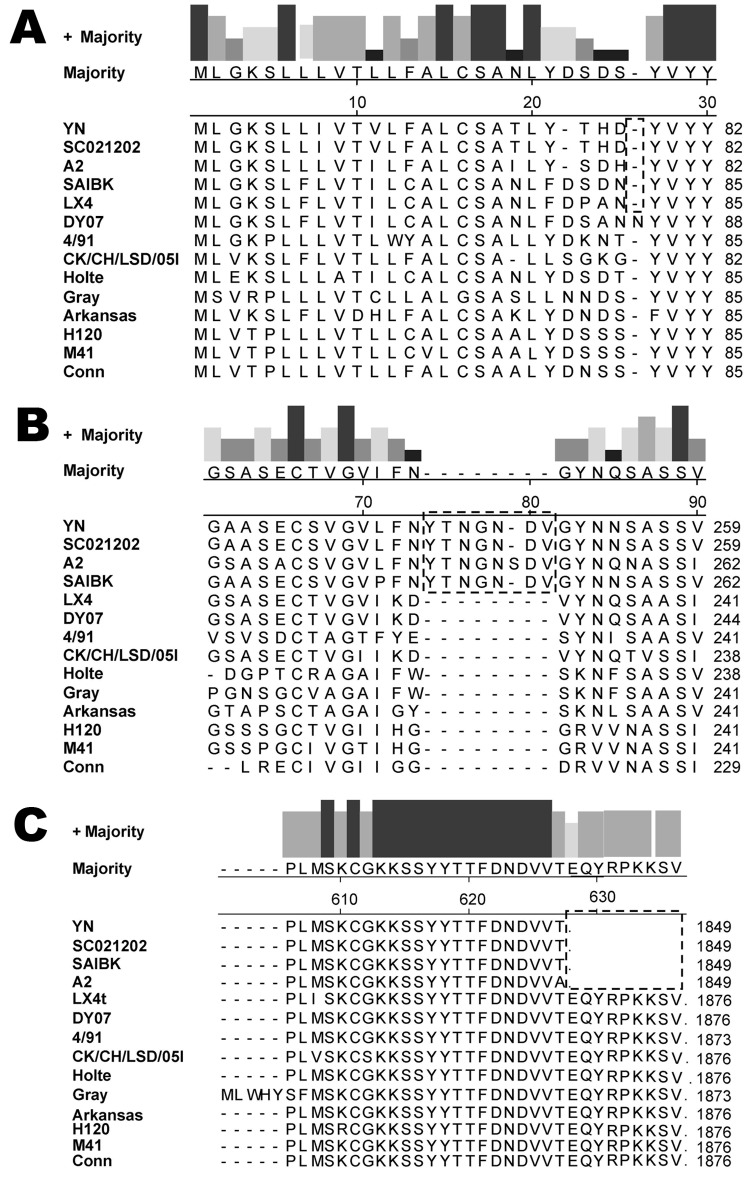
Amino acid sequence alignment for the S1 gene (A, B) and S2 gene (C) of 14 strains of avian infectious bronchitis virus. Strains YN, SC021202, A2, and SAIBK have a 7-aa insertion in the S1 gene and a 9-aa deletion in the S2 gene, compared with most strains.

The S glycoprotein is the major functional protein for IBV; thus, the putative differences between the YN strain and other IBVs were investigated. Analyses showed that a single amino acid insertion was present at position 22 (Figure 2, panel A) in addition to a 7-aa insertion at positions 74–81 (Figure 2, panel B) in the S1 gene of the YN isolate, in which a putative N-glycosylation site was found. Among available sequences in GenBank, strains A2, BJ, CQ04–1, SC021202, and SAIBK had the same insertion in the S1 gene. Compared with other available strains, 9-aa deletions were located at the N terminal of the S2 gene (Figure 2, panel C) from a base transition (G→T) at position 1,849 nt, resulting in premature stop codon in the open reading frame.

### Clinicopathologic Assessment in Chickens

**Figure 3:**
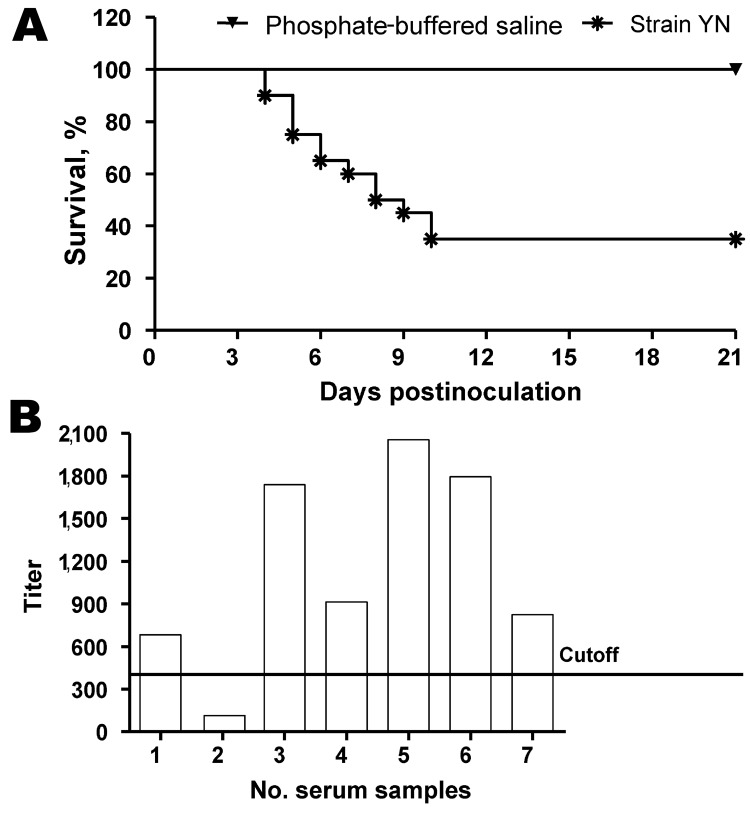
Seroconversion and percentage survival of chickens experimentally infected with infectious bronchitis virus (IBV), People’s Republic of China. A) Survival of chickens after inoculation with IBV YN strain. B) Detection of IBV antibodies by ELISA at 21 days postinoculation. Cutoff titer = 396.

Clinically, some birds appeared depressed with ruffled feathers; these birds had increased water intake and huddled together in YN-inoculation group at 2 days postinfection (dpi). At 4 dpi, IB-like signs became obvious in all infected chickens, and some birds were euthanized because of severe disease. Deaths occurred until 21 dpi; the death rate reached 65% (Figure 3, panel A). No deaths or clinical signs were observed in the control group.

**Figure 4:**
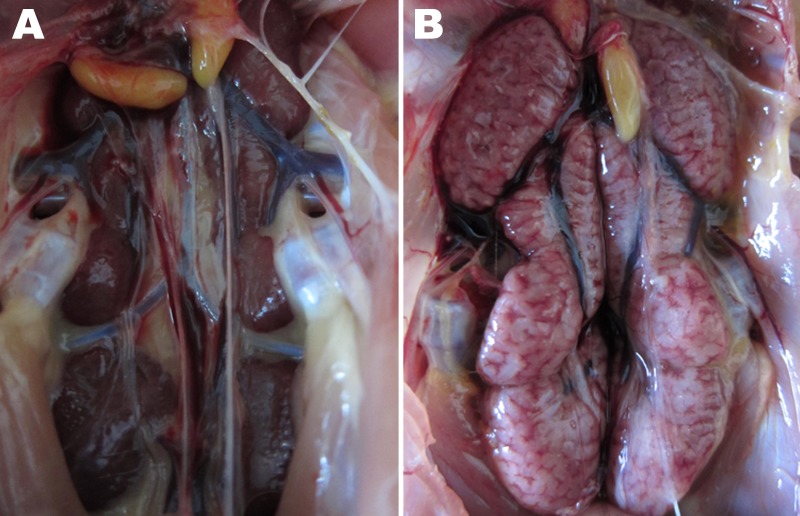
Gross lesions from kidney tissues from chickens experimentally infected with infectious bronchitis virus (IBV). A) Kidney tissue of an uninfected control chicken. B) Obvious enlargement and urate deposition in the kidney of a chick infected with the IBV YN strain at 7 days postinfection.

Slight hemorrhage with serous catarrhal exudate could be seen in the trachea of all euthanized birds. Air sac lesions also were present, characterized by marked thickening of the air sac wall and a yellow caseous exudate. All euthanized chickens showed typical kidney lesions. The affected kidneys were substantially enlarged, and deposits of pale urate were frequently observed in the tubules and ureters (Figure 4). No gross lesions were observed in any birds in the control group.

Antibody responses in birds that survived infection were measured by a commercial ELISA kit (IDEXX Laboratories). All but 1 chicken showed a positive reaction (Figure 3, panel B) by ELISA, and the mean titer induced by the YN strain was 1,250.35 at 21 dpi.

### RT-PCR and Virus Isolation on Tissues

To further examine the pathogenicity of IBV YN, the tissue tropism of the strain was investigated by RT-PCR and virus isolation by using tissue suspensions from dead birds. YN strain replicated well in the various organs, including the kidneys, trachea, lungs, and bursa (Table 3; Technical Appendix).

**Table 3:** Tissue tropism of YN strain of avian IBV, People’s Republic of China*

Method	Tissue examined†					
	Trachea	Lung	Kidney	Bursa	Brain	Liver
RT-PCR‡	10/10	10/10	8/10	7/10	1/10	1/10
Virus isolation§	7/10	8/10	10/10	9/10	ND	ND

*Thirty-day-old SPF chickens were inoculated intranasally with YN IBV (10^5^ 50% egg infectious dose per bird). IBV, infectious bronchitis virus; SPF, specific-pathogen free; RT-PCR, reverse transcription PCR; ND, not detected. †Ten tissue samples from euthanized birds were randomly collected and examined. ‡RT-PCR was directly performed on collected tissues. §Tissue samples were collected for re-isolation of the challenge virus using SPF embryos. Data are the number of tissues from which the virus was isolated/number of tissues examined.

### Histopathologic and IIHC Analyses

**Figure 5:**
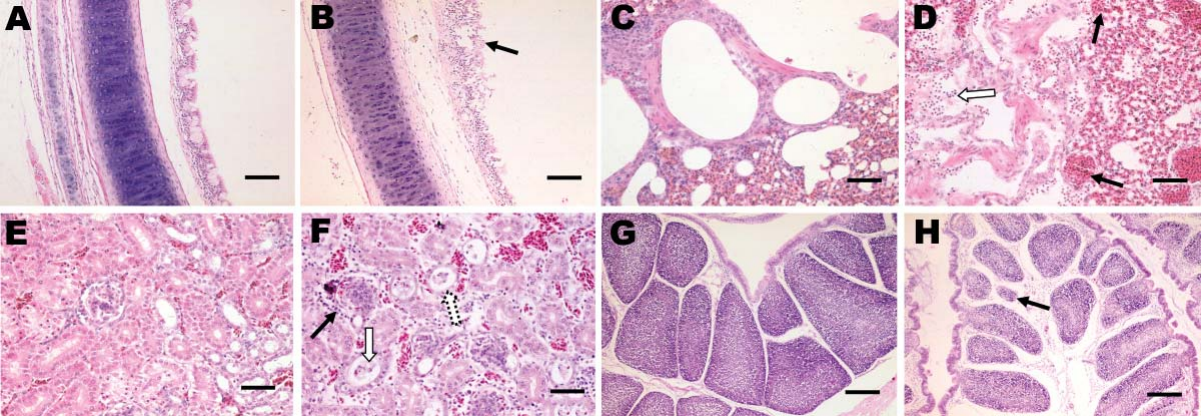
Histopathologic analysis (hematoxylin and eosin stain) of tissues from 30-day-old chickens infected with infectious bronchitis virus YN strain. Panels A, C, E, and G, correspond to control tissues. B) Trachea, extensive dropout, degeneration, and necrosis of the ciliated epithelial cells (black arrow). Scale bar = 100 μm. D) Lung tissue with hemorrhage (black arrow), congestion, and lymphocytic infiltration in alveolar lumen (white arrow). Scale bar = 50 μm. F) Kidney tissue with severe renal lesions, including degeneration (white arrow), and necrosis of renal tubular epithelial cells, lymphocytic infiltration in the interstitium (black arrow), exfoliated renal tubular epithelial cells and erythrocytes were observed extensively. Scale bar = 50 μm. H) Bursa tissue with serous atrophy of lymphoid follicles and widening of the interstitium were observed in bursa of Fabricius (black arrow). Scale bar = 200 μm.

**Figure 6:**
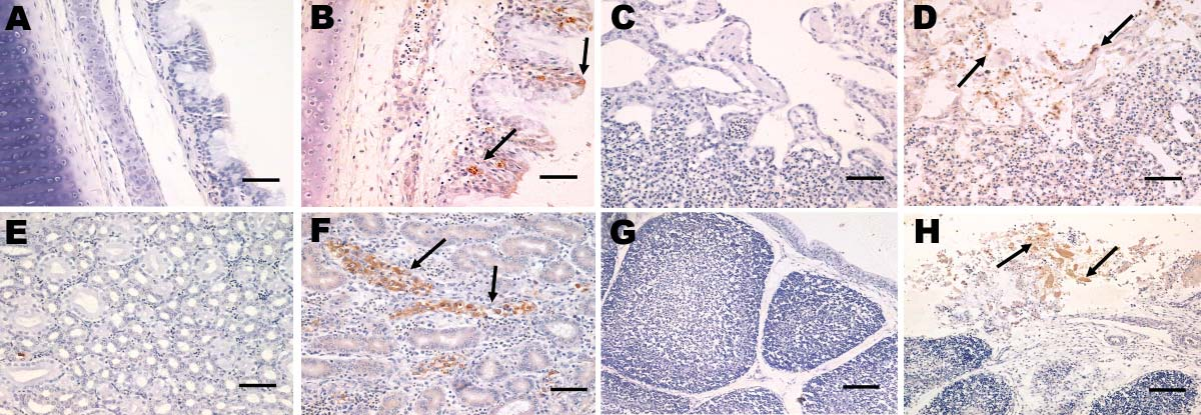
Immunohistochemical detection of avian infectious bronchitis virus (IBV) antigens in tissues after experimental infection with IBV YN strain. Panels A, C, E, and G correspond to control tissues. B) Tracheal tissue with viral antigen detected extensively in the epithelial cells of the tracheal mucosa (black arrow). Scale bar = 50 μm. D) Lung tissue with viral antigen detected in alveolar cells (black arrow). Scale bar = 50 μm. F) Kidney tissue with viral antigens detected widely in the renal tubular epithelial cells (black arrow). Scale bar = 50 μm. H) Bursa tissue with viral antigens detected at high levels in mucosal epithelium in the bursa of Fabricius (black arrow). Scale bar = 100 μm.

Microscopic examination of the tracheal tissues revealed extensive degeneration and necrosis of the ciliated epithelial cells, sometimes with pseudoacinar structures resulting from dropout of dead cells (Figure 5, panel B). Viral antigen was detected at high levels in the epithelial cells of the tracheal mucosa (Figure 6, panel B). Lung lesions were characterized by hemorrhage, congestion, and lymphocytic infiltration in the alveolar lumen (Figure 5, panel D), and viral antigen was detected in alveolar cells (Figure 6, panel D). Severe renal lesions, including degeneration and necrosis of renal tubular epithelial cells, lymphocytic infiltration in the interstitium, exfoliated renal tubular epithelial cells, and erythrocytes were frequently observed (Figure 5, panel F). Viral antigens were detected extensively in the renal tubular epithelial cells (Figure 6, panel F). Serious atrophy of lymphoid follicles and widening of the interstitium were observed in the bursa of Fabricius (Figure 5, panel H), and viral antigens were detected widely in the mucosal epithelium of the bursa of Fabricius (Figure 6, panel H). Sporadic congestion also was observed in the liver and cerebrum.

## Discussion

IBV infection leads to severe economic losses in the poultry industry because of poor weight gain and feeding efficiency in broilers or reduced egg production and egg quality in laying birds. Death rates are often low (<30%) unless secondary bacterial infections cause increased deaths of birds (*21*,*22*). Evaluation of the pathogenicity of IBV was based on several criteria, including: clinical signs, gross pathologic lesions, histopathologic changes, and tissue tropism. In this study, a China IBV strain isolated from a 30-day-old vaccinated broiler flock that had a history of respiratory signs, severe renal disease, and higher than expected mortality rate was analyzed by clinical observation, histopathologic examination, RT-PCR, virus isolation, and immunohistochemistry. Analyses revealed that the YN strain induced severe pathogenicity in 30-day-old SPF chickens with a 65% death rate and substantial kidney lesions.

Sequence analysis of the S1 gene of YN isolate showed high nucleotide similarities to CQ04–1 (99.5% nt and 98.8% aa identities) and SC021202 (99.3% nt and 98.6% aa identities). Phylogenetic analysis further indicated that at least 3 distinct genetic clusters of IBV were present in chicken flocks in China, named A2-like, LSD-like, and M41-like strains. Most China IBV field isolates belonged to the same genetic cluster (A2-like), but the serotypes of these prevalent IBV strains differed from those of the currently used vaccine strains in China (M41-like, e.g., H120, Ma5, 28/86, and W93), which are considered the main cause of disease outbreaks (*5*,*15*–*17*). An attenuated or inactivated vaccine that matches with pandemic strains is urgently needed in China to control IBV infection.

IBV has been shown to replicate in many respiratory tissues (including trachea, lungs, and air sac), causing respiratory disease; in some urogenital tissues (including kidney), causing minor or major nephritis; and in many parts of the alimentary tract (including esophagus, proventriculus, and intestine) (*23*–*25*). As expected, YN was most often detected in the trachea, lung, kidney, and bursa by RT-PCR, virus isolation, and IHC analysis. In addition, we found YN antigens partly from liver (1/10) and brain (1/10).

Among 4 major structural proteins, the S glycoprotein is known to contain regions that induce neutralizing, serotype-specific, membrane fusion, attachment, and hemagglutination-inhibiting antibodies (*5*,*26*–*28*). In addition, the S protein is a determinant of cell tropism (*29*,*30*). Our data showed a 7-aa insertion between positions 72 and 78 (Figure 2, panel A) in the S1 gene of the YN strain and a 9-aa deletion in the N terminal of the S2, generating a stop codon. These changes are possibly related to the increased virulence observed for YN IBV, and reverse genetic analyses are needed to confirm these findings.

In conclusion, we showed that YN-like viruses are a predominant strain in China and that YN IBV exhibits broad tissue tropism and severe pathogenicity in chickens. To better control IBV in China, more detailed analysis of the biologic and antigenic characteristics of the predominant IBV isolates is warranted, and assessments of the efficacy of current vaccines against these isolates are needed.

Technical Appendix Results of reverse transcription PCR analysis of tissues from chickens experimentally infected with infectious bronchitis virus.
