# Supplementary material for: Virulent Avian Infectious Bronchitis Virus, People’s Republic of China
Source: Emerg Infect Dis. 2012 Dec;18(12):1994–2001. doi: 10.3201/eid1812.120552 (PMC3557894; doi:10.3201/eid1812.120552)
Supplement: Technical Appendix — Results of reverse transcription PCR analysis of tissues from chickens experimentally infected with infectious bronchitis virus. [file 12-0552-Techapp-s1.pdf]

Article DOI: http://dx.doi.org/10.3201/eid1812.120552

Virulent Avian Infectious Bronchitis Virus, People's Republic of China

Technical Appendix

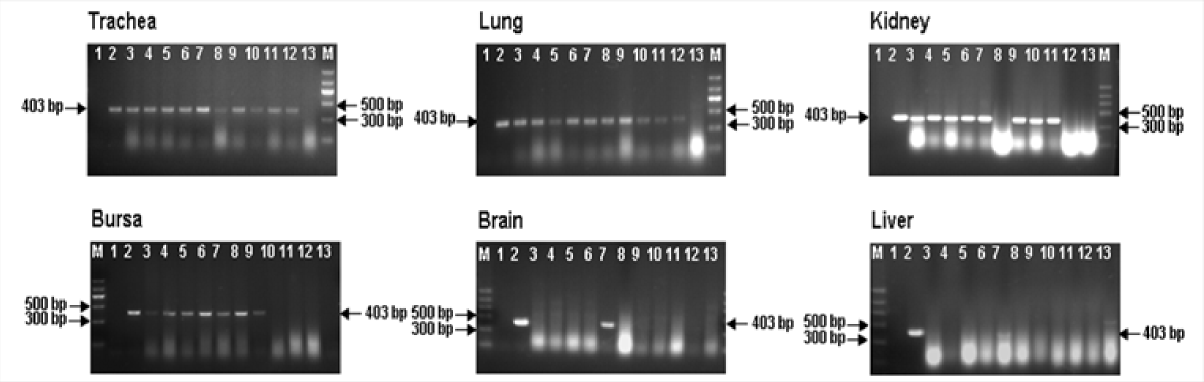

Technical Appendix Figure. Results of reverse transcription PCR analysis of tissues from chickens experimentally infected with infectious bronchitis virus (IBV). Lane 1, water; lane 2, reference positive control (IBV); lanes 3–12, tissues from birds infected with IBV YN strain; lane 13, normal tissue, lane M, DNA marker.
